# 5-HT_2A_ receptor activation induces CB_1_-dependent presynaptic inhibitory long-term depression in the nucleus accumbens core

**DOI:** 10.3389/fphar.2026.1916522

**Published:** 2026-10-07

**Authors:** Camila Estay-Olmos, Koyam Morales-Weil, Juan Ahumada, Ramón Sotomayor-Zárate, Marco Fuenzalida

**Affiliations:** 1 Centro de Neurobiología y Fisiopatología Integrativa (CENFI), Facultad de Ciencias, Universidad de Valparaíso, Valparaíso, Chile; 2 Departamento de Ciencias Biomédicas, Facultad de Ciencias de la Vida, Universidad Viña del Mar, Viña del Mar, Chile; 3 Millennium Nucleus of Neuroepigenetics and Plasticity (EpiNeuro), Universidad de Valparaiso, Santiago, Chile; 4 Centro Interdisciplinario de Neurociencia de Valparaíso (CINV), Facultad de Ciencias, Universidad de Valparaíso, Valparaíso, Chile; 5 Facultad de Ciencias, Instituto de Fisiología, Universidad de Valparaíso, Valparaíso, Chile

**Keywords:** 5-HT_2A_ receptor, CB_1_ receptor, GABA_A_ receptor, iLTD, nucleus accumbens

## Abstract

The serotonin 2A receptor (5-HT_2A_) is a G_q/11_-coupled receptor implicated in reward processing, neuropsychiatric disorders, and the actions of serotonergic psychedelics. Although 5-HT_2A_ activation has been shown to engage endocannabinoid (eCB) signaling in several brain regions, its role in inhibitory synaptic plasticity within the nucleus accumbens (NAc) remains poorly understood. Here, we investigated whether 5-HT_2A_ activation modulates GABAergic synaptic transmission onto medium spiny neurons (MSNs) in the mouse NAc core. Using whole-cell patch-clamp recordings, we found that activation of 5-HT_2A_ receptors with the selective agonist TCB-2 induced a robust inhibitory long-term depression (iLTD) of evoked GABAergic synaptic transmission. This depression was accompanied by reduced spontaneous and miniature IPSC frequency, increased paired-pulse ratio, and changes in coefficient of variation analysis consistent with a presynaptic reduction in GABA release probability. Mechanistically, iLTD was occluded by the CB_1_ receptor agonist WIN55,212-2 and blocked by the CB_1_ receptor antagonist AM251, supporting a critical role for endocannabinoid signaling. Furthermore, iLTD induction required postsynaptic G-protein signaling, phospholipase Cβ (PLCβ) activity, and synthesis of the endocannabinoid 2-arachidonoylglycerol (2-AG). Together, these findings demonstrate that 5-HT_2A_ activation induces a CB_1_-dependent presynaptic iLTD through a postsynaptic PLCβ/2-AG signaling cascade. These results demonstrate that 5-HT_2A_ receptor activation engages endocannabinoid signaling to regulate inhibitory synaptic transmission in the NAc, highlighting a mechanism through which 5-HT_2A_ signaling may modulate mesolimbic circuit function.

## 1 Introduction

The serotonin 2A receptor (5-HT_2A_) is a G_q/11_-coupled receptor that plays a central role in serotonergic neuromodulation and mediates many of the cellular and behavioral effects of serotonergic psychedelics (Aghajanian and Marek, 1999; Carhart-Harris et al., 2016; Nichols, 2016). Activation of 5-HT_2A_ engages intracellular signaling pathways involving phospholipase Cβ (PLCβ) and diacylglycerol production, enabling modulation of synaptic transmission across multiple brain regions (Parrish and Nichols, 2006; Béïque et al., 2007; Meza et al., 2026). Dysregulation of 5-HT_2A_ signaling has been implicated in the pathophysiology of several neuropsychiatric disorders, including major depressive disorder, anxiety disorders, and substance use disorders (Nichols, 2016; Fryc et al., 2025).

The nucleus accumbens (NAc) is a key component of the mesolimbic reward circuit and plays a central role in motivated behavior, reinforcement learning, and affective processing (Koob and Volkow, 2010). Both NAc function and 5-HT_2A_ signaling have been implicated in neuropsychiatric disorders, including major depressive disorder and substance use disorders (Dayan and Huys, 2009; Koob and Volkow, 2010). A recent single-cell transcriptomic and *in situ* hybridization study identified Htr2a expression in striatal medium spiny neurons, supporting a potential direct role for 5-HT_2A_ receptor signaling in modulating their function (Cardozo Pinto et al., 2026). Previous studies have shown that 5-HT_2A_ activation can engage retrograde endocannabinoid (eCB) signaling to modulate synaptic transmission across distinct brain regions (Best and Regehr, 2008; Burattini et al., 2014; Meza et al., 2026). For example, activation of 5-HT_2A_ suppresses excitatory transmission in the inferior olive and inhibitory transmission in the prefrontal cortex through mechanisms involving endocannabinoid release and presynaptic CB_1_ receptor activation (Best and Regehr, 2008). In the NAc, endocannabinoid (eCB)-dependent long-term depression has been extensively characterized at excitatory synapses (Burattini et al., 2014), underscoring the critical role of eCB signaling in the regulation of accumbal synaptic plasticity. However, whether activation of 5-HT_2A_ engages eCB mechanisms to modulate inhibitory synaptic transmission within the NAc remains unexplored. In the present study, we examined whether activation of 5-HT_2A_ modulates inhibitory synaptic transmission onto medium spiny neurons (MSNs) in the NAc core. Using whole-cell patch-clamp recordings and pharmacological approaches, we found that 5-HT_2A_ activation induces a presynaptically expressed inhibitory long-term depression (iLTD) of GABAergic transmission. This form of plasticity requires CB_1_ receptor activation, postsynaptic G-protein signaling, phospholipase Cβ (PLCβ) activity, and synthesis of the endocannabinoid 2-arachidonoylglycerol (2-AG). These findings identify a previously unrecognized interaction between serotonergic and endocannabinoid signaling pathways in the NAc and reveal a novel mechanism through which 5-HT_2A_ regulates inhibitory synaptic transmission.

## 2 Materials and methods

### 2.1 Drugs and pharmacological reagents

The following pharmacological agents were used in this study: serotonin hydrochloride (5-HT; Tocris Bioscience, Cat. No. 3547), D-(-)-2-amino-5-phosphonopentanoic acid (D-AP5; Tocris Bioscience, Cat. No. 0106), 6-cyano-7-nitroquinoxaline-2,3-dione disodium salt (CNQX; Tocris Bioscience, Cat. No. 1045), TCB-2 (Tocris Bioscience, Cat. No. 2592), AM251 (Tocris Bioscience, Cat. No. 1117), WIN55, 212-2 (Cayman Chemical, Item No. 10009023), MDL100907 (Tocris Bioscience, Cat. No. 4173), U73122 (Tocris Bioscience, Cat. No. 1268), RHC80267 (Tocris Bioscience, Cat. No. 1842), guanosine 5′-[β-thio]diphosphate trilithium salt (GDPβS; Sigma-Aldrich, CAS 97952-36-8) and tetrodotoxin citrate (TTX; Cayman Chemical, Item No. 14964). The concentrations used, along with the reported pharmacological affinity or potency, selectivity, and supporting references for each compound, are summarized in Supplementary Table S1(Araneda and Andrade, 1991; Austgen and Kline, 2013; Choi et al., 2005; Morales-Weil et al., 2020; Santander et al., 2025; Zhang, 2003; Zhong et al., 2015).

### 2.2 Animals

Male C57BL/6J mice (postnatal days 30–50) were used in this study and housed in the animal facility of the Neuronal Plasticity Laboratory, Institute of Physiology, Universidad de Valparaíso. Animals were maintained under controlled temperature (22 °C ± 2 °C) and humidity (55% ± 5%), on a 12 h light/dark cycle, with food and water available *ad libitum*.

### 2.3 Decapitation and brain extraction

Animals were anesthetized with isoflurane (3%–5% in oxygen) delivered through a calibrated vaporizer until an adequate depth of anesthesia was achieved, as confirmed by the absence of reflex responses. Animals were then euthanized by decapitation using a small-animal guillotine (model 51330, Stoelting Co., Wood Dale, IL, USA). Brains were rapidly removed and used for subsequent experiments.

### 2.4 Slice preparation

Following decapitation, brains were quickly extracted and immersed in ice-cold (0 °C–2 °C) artificial cerebrospinal fluid (ACSF) containing (in mM): 124 NaCl, 2.7 KCl, 1.25 KH_2_PO_4_, 2 MgSO_4_, 26 NaHCO_3_, 2.5 CaCl_2_, and 10 glucose (pH 7.4), continuously bubbled with carbogen (95% O_2_, 5% CO_2_). Coronal brain slices (300 µm thick) were prepared using a vibrating microtome (Vibroslice VSL; World Precision Instruments, Sarasota, FL, USA).

Slices were incubated in oxygenated ACSF for at least 1 h at room temperature before electrophysiological recordings. For recordings, slices were transferred to an immersion-type recording chamber (1 mL) mounted on an upright microscope (FN100 IR; Nikon, Tokyo, Japan) equipped with infrared differential interference contrast (IR-DIC) optics and a ×40 water-immersion objective. During recordings, slices were continuously perfused with carbogenated aCSF at a flow rate of 2 mL·min^-1^ and maintained at room temperature (22 °C).

### 2.5 Electrophysiology

Whole-cell patch-clamp recordings were performed in voltage-clamp mode to record inhibitory postsynaptic currents (IPSCs). Neurons located in the core region of the nucleus accumbens (NAc) were voltage-clamped at 0 mV. Patch pipettes were sealed onto visually identified neurons, and recordings were initiated after achieving a high-resistance seal (>1 GΩ) and establishing whole-cell configuration. Recording electrodes consisted of Ag/AgCl wires inserted into borosilicate glass pipettes filled with the following intracellular solution: Cesium-based internal solution (Cs-gluconate): (in mM) 131 Cs-gluconate, 10 HEPES, 10 EGTA, 2 glucose, 1 CaCl_2_, 8 NaCl, 4 Mg_2_ATP, and 0.4 Na_3_GTP buffered to pH 7.2–7.3 with CsOH (Guiffa-Gómez et al., 2026; Meza et al., 2026). Evoked IPSCs were elicited using a bipolar stimulating electrode positioned within the NAc core, approximately 100 µm from the recorded neuron and oriented toward the soma.

### 2.6 Patch-clamp recording analysis

After positioning the recording and stimulation electrodes (Figure 1A), visually identified medium spiny neurons (MSNs) were patched in whole-cell voltage-clamp configuration and held at 0 mV to record inhibitory synaptic currents. Following break-in, cells were allowed to stabilize for at least 5 min to permit intracellular dialysis. Baseline responses were then recorded for 10 min, after which the appropriate pharmacological agent was applied via bolus. All recordings were performed in the continuous presence of the glutamatergic receptor antagonists APV (25 µM) and CNQX (20 µM) to isolate inhibitory postsynaptic currents (IPSCs). The recorded eIPSCs were abolished by picrotoxin (PTX, 50 μM; Supplementary Figures S1A,B), confirming their mediation by GABA_A_ receptors.

**FIGURE 1:**
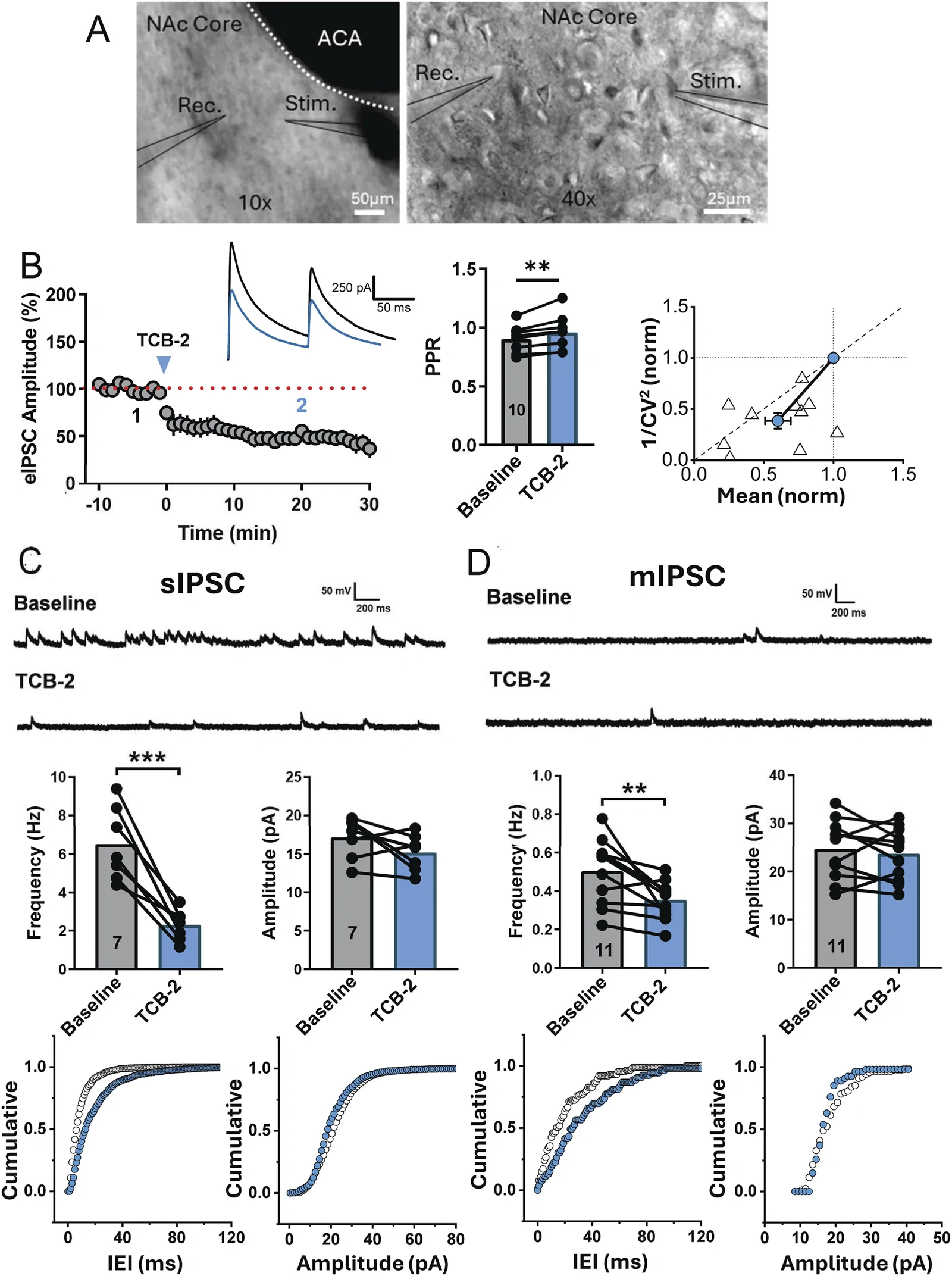
Activation of 5-HT_2A_ receptors induces a presynaptically expressed iLTD of inhibitory transmission in the NAc core. **(A)** Representative images showing the recording configuration in the nucleus accumbens (NAc) core. Recording and stimulation electrodes were positioned within the NAc core to evoke IPSCs in MSNs. **(B)** Time course of normalized eIPSCs amplitude showing the effect of the selective 5-HT_2A_ agonist TCB-2 (10 μM, 1 min). Insets show representative eIPSC traces recorded at baseline (black) and 20 min after TCB-2 application (blue). Summary plots show paired-pulse ratio (PPR) and CV analysis (1/CV^2^ versus mean amplitude). **(C)** Representative traces and summary data of sIPSCs recorded before and after TCB-2 application. TCB-2 reduced sIPSC frequency without significantly affecting event amplitude. Bottom panels show cumulative probability distributions of inter-event intervals and amplitudes. **(D)** Representative traces and summary data of mIPSCs recorded in the presence of TTX. TCB-2 reduced mIPSC frequency without altering event amplitude. Bottom panels show cumulative probability distributions of inter-event intervals and amplitudes. Data are presented as mean ± SEM. Bar graphs show individual cells connected by lines. Statistical significance was assessed using paired Student’s t-tests. **P < 0.01; ***P < 0.001.

Evoked IPSCs (eIPSCs) were elicited using a paired-pulse stimulation protocol consisting of two electrical stimuli delivered 100 ms apart. To assess potential changes in presynaptic neurotransmitter release probability, the paired-pulse ratio (PPR) was calculated as the ratio of the amplitude of the second response to that of the first response (PPR = R_2_/R_1_). PPR values were determined from responses averaged over the last 5 min of baseline and over a 5-min period beginning 20 min after treatment application. As an additional measure of presynaptic function, the coefficient of variation (CV) of eIPSC amplitudes was calculated before and after pharmacological manipulations. CV was computed as the standard deviation divided by the mean amplitude of a series of evoked responses obtained during the analysis period. To assess potential changes in release probability, data were plotted as normalized mean eIPSC amplitude (M) versus 1/CV^2^ (Brock et al., 2020; Guiffa-Gómez et al., 2026). The stability of synaptic responses throughout the recording period was assessed in separate time-control experiments, in which ACSF was applied at the time point corresponding to pharmacological treatment. eIPSC amplitude and PPR were analyzed over the same baseline and post-application time windows described above (n = 6 cells from 2 mice; Supplementary Figures S1C–E).

### 2.7 Statistical analysis

Electrophysiological data were analyzed using Clampfit (pCLAMP 10.7; Molecular Devices, USA). Processed data were exported to OriginLab (version 10.6) for graphical representation and to GraphPad Prism (version 9.0) for statistical analysis. Data normality was assessed using the Shapiro–Wilk test. For comparisons between two conditions, two-tailed paired Student’s *t* tests were performed. When appropriate, two-way ANOVA was conducted to compare multiple conditions, followed by Holm–Sidak *post hoc* test. Statistical significance was set at *P* < 0.05. Data are presented as mean ± SEM unless otherwise stated. For electrophysiological experiments, n represents the number of recorded neurons (one neuron per NAc slice), while the number of mice used is reported separately.

## 3 Results

To determine the effect of 5-HT_2A_ receptor activation on local inhibitory synaptic transmission onto MSNs in the NAc, we recorded evoked IPSCs from MSNs held at 0 mV in the continuous presence of the ionotropic glutamatergic receptor antagonists APV and CNQX (Figure 1A). We observed that a brief application of TCB-2 (10 μM final concentration, 1 min) induced an iLTD of evoked inhibitory postsynaptic currents (eIPSCs). TCB-2 was delivered as a bolus into the recording chamber and subsequently washed out by continuous ACSF perfusion. eIPSC amplitude significantly decreased 20 min after drug application compared with baseline (Baseline: 97.15% ± 1.57%; TCB-2: 49.61% ± 6.28%; *P* < 0.0001, n = 10 cells from 5 mice; Figure 1B). This iLTD was accompanied by a significant increase in PPR (baseline: 0.90 ± 0.03; TCB-2: 0.96 ± 0.04, *P* = 0.015, n = 10 cells from 5 mice), and by changes in the CV expressed as 1/CV^2^ of synaptic responses (slope: 1.127 ± 0.252, *P* = 0.002, n = 10 cells from 5 mice; Figure 1B). These results suggest that activation of 5-HT_2A_ receptors induces a decrease in the probability of GABA release.

To further examine the locus of expression of 5-HT_2A_-induced iLTD, we analyzed spontaneous IPSCs (sIPSCs) following TCB-2 application. Recordings of sIPSCs revealed a significant reduction in event frequency after TCB-2 application (Baseline: 6.513 ± 0.7234 Hz; TCB-2: 2.292 ± 0.302 Hz; *P* = 0.0009, n = 7 cells from 3 mice; Figure 1C), whereas sIPSC amplitude remained unchanged (Baseline: 17.13 ± 1.009 pA; TCB-2: 15.18 ± 0.8947 pA; *P* = 0.1392, n = 7 cells from 3 mice; Figure 1C). These findings are consistent with a presynaptic reduction in GABA release. To determine whether this effect persisted in the absence of action potential-dependent synaptic activity, miniature IPSCs (mIPSCs) were recorded in the presence of tetrodotoxin (TTX, 500 nM). Similary, TCB-2 significantly reduced mIPSC frequency (Baseline: 0.504 ± 0.05 Hz; TCB-2: 0.35 ± 0.03 Hz; *P* = 0.0078, n = 11 cells from 3 mice; Figure 1D) without affecting mIPSC amplitude (Baseline: 24.69 ± 1.859 pA; TCB-2: 23.67 ± 1.647 pA; *P* = 0.4759, n = 11 cells from 3 mice; Figure 1D). Consistent with these findings, cumulative probability distributions revealed pronounced shifts in inter-event interval distributions for both sIPSCs (K–S test, D = 0.311, *P* < 0.0001) and mIPSCs (K–S test, D = 0.465, *P* < 0.0001) (Figures 1C,D). Together with the increase in paired-pulse ratio and changes in CV analysis, these results support a predominantly presynaptic locus of expression for 5-HT_2A_-induced iLTD. To determine whether the observed iLTD could also be induced by serotonin (5-HT), we examined the effects of exogenous 5-HT application on inhibitory transmission in NAc MSNs. Brief application of 5-HT (10 μM) significantly reduced eIPSC amplitude (Baseline: 97.05% ± 1.79%; 5-HT: 71.44% ± 4.14%; *P* = 0.0008, n = 7 cells from 3 mice; Supplementary Figures S2A–C). This effect was prevented by the selective 5-HT_2A_ antagonist MDL100907 (1 μM) (Baseline: 98.59% ± 1.84%; 5-HT: 94.26% ± 11.95%; *P* = 0.72, n = 7 cells from 4 mice; Supplementary Figures S2D–F), confirming that serotonin-induced depression of inhibitory transmission is mediated by 5-HT_2A_.

Given the presynaptic locus of expression of 5-HT_2A_-induced iLTD and previous evidence linking 5-HT_2A_ activation to endocannabinoid signaling in other brain regions, we next examined whether CB_1_ receptor activation contributes to this form of plasticity. Bath application of the CB_1_ receptor agonist WIN55,212-2 (WIN; 5 μM) produced a robust depression of eIPSC amplitude (Baseline: 99.0% ± 2.64%; WIN: 53.13% ± 7.59%; *P* = 0.0003, n = 6 cells from 5 mice; Figures 2A,B) and increased paired-pulse ratio (Baseline: 0.79 ± 0.13; WIN: 0.93 ± 0.11; *P* = 0.0098, n = 6 cells from 5 mice; Figure 2C), consistent with a reduction in presynaptic GABA release probability. To determine whether 5-HT_2A_-induced iLTD and CB_1_ receptor activation engage a common signaling pathway, TCB-2 was applied in the continuous presence of WIN. Under these conditions, TCB-2 failed to induce additional depression of eIPSC amplitude (Baseline: 99.28% ± 2.01%; TCB-2: 88.17% ± 6.03%; *P* = 0.06, n = 6 cells from 4 mice; Figures 2D,E) and did not alter PPR (Baseline: 0.80 ± 0.06; TCB-2: 0.86 ± 0.06; *P* = 0.11, n = 6 cells from 4 mice; Figure 2F), indicating occlusion of 5-HT_2A_-mediated iLTD. Likewise, the CB_1_ receptor antagonist/inverse agonist AM251 (10 μM) abolished the effects of TCB-2 on both eIPSC amplitude (Baseline: 96.6% ± 2.6%; TCB-2: 98.3% ± 10.9%; *P* = 0.89, n = 8 cells from 5 mice; Figures 2G,H) and PPR (Baseline: 0.94 ± 0.07; TCB-2: 0.90 ± 0.04; *P* = 0.84, n = 8 cells from 5 mice; Figure 2I). Together, these findings indicate that activation of CB_1_ receptors is required for the induction of 5-HT_2A_-dependent iLTD and suggest that both manipulations converge on a common presynaptic mechanism regulating GABA release.

**FIGURE 2:**
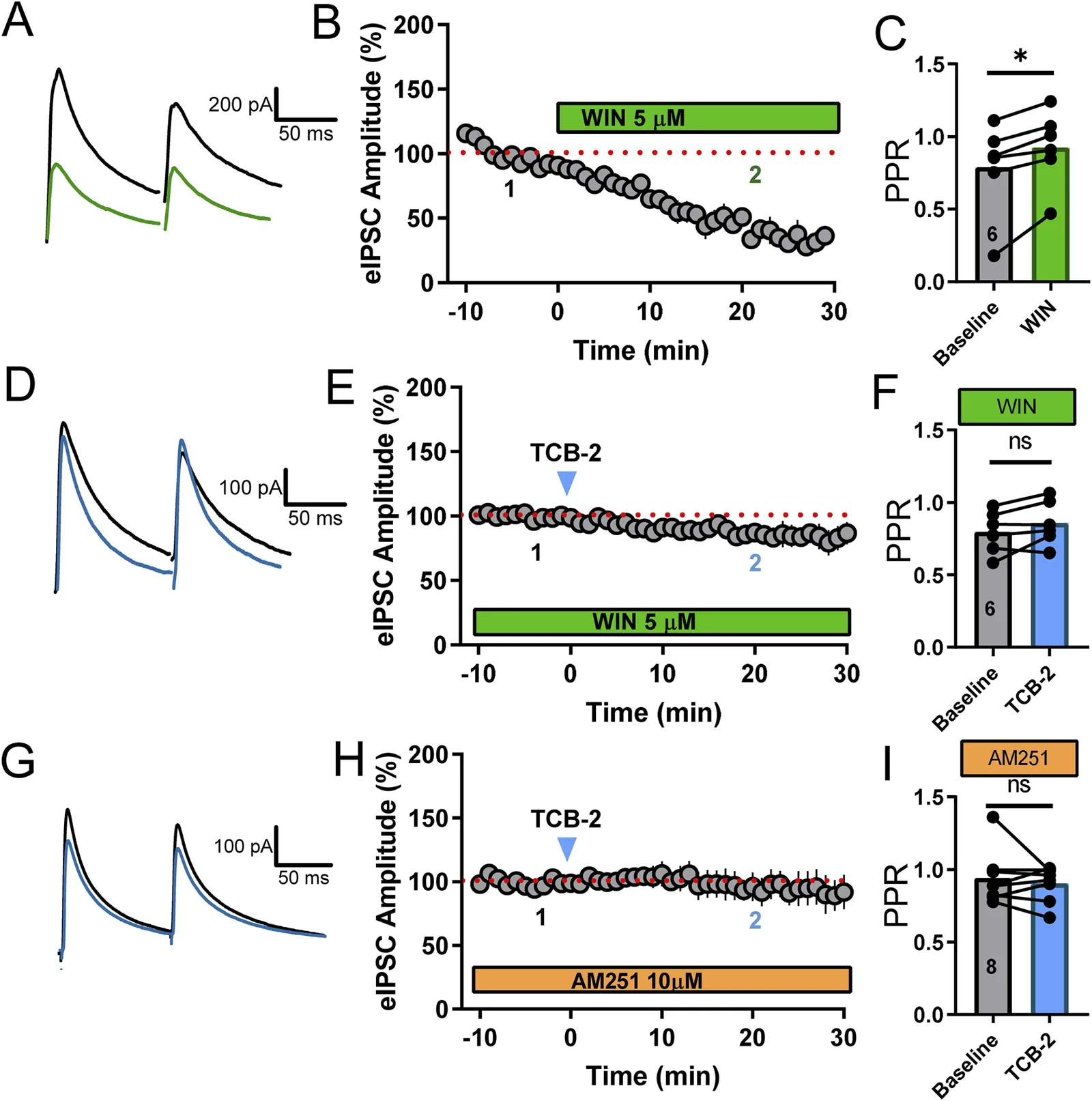
5-HT_2A_-induced iLTD requires presynaptic CB_1_ receptor activation. **(A)** Representative eIPSC traces recorded before (black) and after (green) application of the CB_1_ receptor agonist WIN55,212-2 (WIN; 5 μM). **(B)** Time course of normalized eIPSC amplitude showing the effect of WIN application. WIN induced a long-lasting depression of inhibitory synaptic transmission. **(C)** Summary plot showing the increase in paired-pulse ratio (PPR) following WIN application, consistent with a reduction in presynaptic GABA release probability. **(D)** Representative eIPSC traces recorded before (black) and after (blue) TCB-2 application in the continuous presence of WIN. **(E)** Time course of normalized eIPSC amplitude showing that prior activation of CB1 receptors by WIN occludes TCB-2-induced iLTD. **(F)** Summary plot showing that TCB-2 fails to alter PPR in the presence of WIN. **(G)** Representative eIPSC traces recorded before (black) and after (blue) TCB-2 application in the presence of the CB1 receptor antagonist/inverse agonist AM251 (10 μM). **(H)** Time course of normalized eIPSC amplitude showing that AM251 prevents TCB-2-induced iLTD. **(I)** Summary plot showing that TCB-2 fails to alter PPR in the presence of AM251. Data are presented as mean ± SEM. Insets show representative traces recorded at baseline and 20 min after drug application. Bar graphs show individual cells connected by lines. Statistical significance was assessed using paired Student’s t-tests. *P < 0.05; n.s., not significant.

To determine whether 5-HT_2A_-induced iLTD requires postsynaptic G protein signaling, we included GDPβS (2 mM), a non-hydrolyzable GDP analog that prevents G-protein activation, in the recording pipette. Under these conditions, TCB-2 failed to significantly alter eIPSC amplitude (Baseline: 104.63% ± 2.70%; TCB-2: 87.57% ± 10.99%; *P* = 0.14, n = 9 cells from 6 mice; Figures 3A,B) or PPR (Baseline: 1.06 ± 0.15; TCB-2: 0.94 ± 0.06; *P* = 0.32, n = 9 cells from 6 mice; Figure 3C), indicating that postsynaptic G-protein signaling is required for iLTD induction. Because 5-HT_2A_ receptors signal primarily through G_q/11_-mediated activation of PLCβ (González-Maeso et al., 2007; Nichols, 2016), we next examined the contribution of this pathway to 5-HT_2A_-induced iLTD. Intracellular application of the PLCβ inhibitor U73122 (20 μM) significantly attenuated TCB-2-induced depression of eIPSC amplitude (Baseline: 93.7% ± 5.3%; TCB-2: 75.9% ± 3.6%; *P* = 0.047, n = 7 cells from 3 mice; Figures 3A,B) and prevented changes in PPR (*P* = 0.64; Figure 3C), suggesting that PLCβ signaling contributes to the induction of iLTD. To determine whether endocannabinoid synthesis is required, we next inhibited diacylglycerol lipase (DAGL), the enzyme responsible for 2-arachidonoylglycerol (2-AG) production. Intracellular application of the DAGL inhibitor RHC80267 (30 μM) abolished the effects of TCB-2 on both eIPSC amplitude (Baseline: 102.0% ± 3.4%; TCB-2: 89.14% ± 5.9%; *P* = 0.40, n = 6 cells from 2 mice; Figures 3A,B) and PPR (Baseline: 0.85 ± 0.06; TCB-2: 0.95 ± 0.10; *P* = 0.26, n = 6 cells from 2 mice; Figure 3C), indicating that 2-araquidonilglicerol (2-AG) synthesis is necessary for the induction of 5-HT_2A_-dependent iLTD. Together, these findings support a model in which activation of postsynaptic 5-HT_2A_ engages a G-protein-dependent signaling cascade involving PLCβ and 2-AG synthesis, leading to retrograde activation of presynaptic CB_1_ receptors and long-term decrease of GABA release.

**FIGURE 3:**
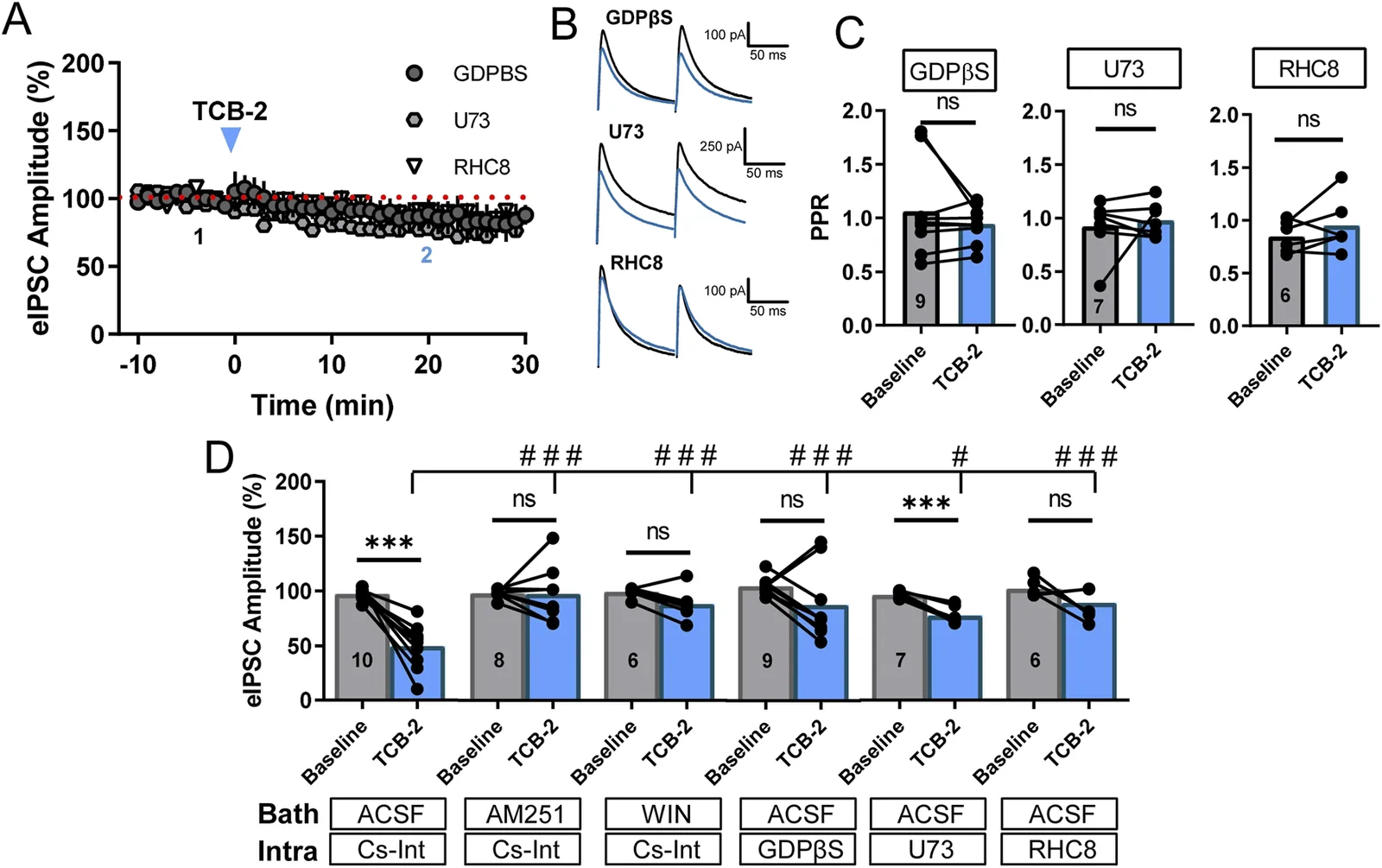
5-HT_2A_-induced iLTD requires postsynaptic G-protein signaling, PLCβ activity, and 2-AG synthesis. **(A)** Time course of normalized eIPSC amplitude following TCB-2 application in neurons dialyzed with GDPβS (2 mM), the PLCβ inhibitor U73122 (20 μM), or in slices treated with the DAG lipase inhibitor RHC80267 (30 μM). **(B)** Representative eIPSC traces recorded at baseline (black) and 20 min after TCB-2 application (blue) for each experimental condition. **(C)** Summary plots showing paired-pulse ratio (PPR) before and after TCB-2 application in the presence of GDPβS, U73122, or RHC80267. Under these conditions, TCB-2 failed to significantly alter PPR. **(D)** Summary of normalized eIPSC amplitudes across all pharmacological conditions. TCB-2-induced iLTD was prevented by blockade of CB1 receptors (AM251), prior activation of CB_1_ receptors (WIN55,212-2), intracellular inhibition of G-protein signaling (GDPβS), inhibition of PLCβ (U73122), or inhibition of diacylglycerol lipase-mediated 2-arachidonoylglycerol (2-AG) synthesis (RHC80267). Data are presented as mean ± SEM. Insets show representative traces recorded at baseline and 20 min after TCB-2 application. Bar graphs show individual cells connected by lines. Within-group comparisons were performed using paired Student’s t-tests and are indicated by asterisks (*P < 0.05, **P < 0.01, ***P < 0.001). Comparisons between experimental conditions were performed using two-way ANOVA followed by Holm–Sidak’s multiple-comparisons test and are indicated by number signs (^#^ *P* < 0.05, ^##^ *P* < 0.01, ^###^ *P* < 0.001). n.s., not significant.

To compare the effects of the different pharmacological manipulations, eIPSC amplitudes were analyzed using two-way ANOVA followed by Holm–Sidak’s multiple-comparisons test. All groups displayed comparable baseline responses, whereas TCB-2-induced iLTD was significantly reduced under AM251 (*P* < 0.0001), WIN55,212-2 (*P* = 0.0004), GDPβS (*P* < 0.0001), U73122 (*P* = 0.0256), and RHC80267 (*P* = 0.0003) relative to control conditions (Figure 3D). These comparisons collectively identify the signaling pathway underlying 5-HT_2A_-induced iLTD, linking postsynaptic G-protein signaling and PLCβ-dependent 2-AG synthesis to retrograde activation of presynaptic CB_1_ receptors.

Collectively, our findings demonstrate that activation of 5-HT_2A_ induces a presynaptically expressed iLTD of inhibitory transmission in the NAc core. This form of plasticity requires postsynaptic G-protein signaling, PLCβ activity, and 2-arachidonoylglycerol (2-AG) synthesis, leading to retrograde activation of presynaptic CB_1_ receptors and a persistent reduction in GABA release (Figure 4).

**FIGURE 4:**
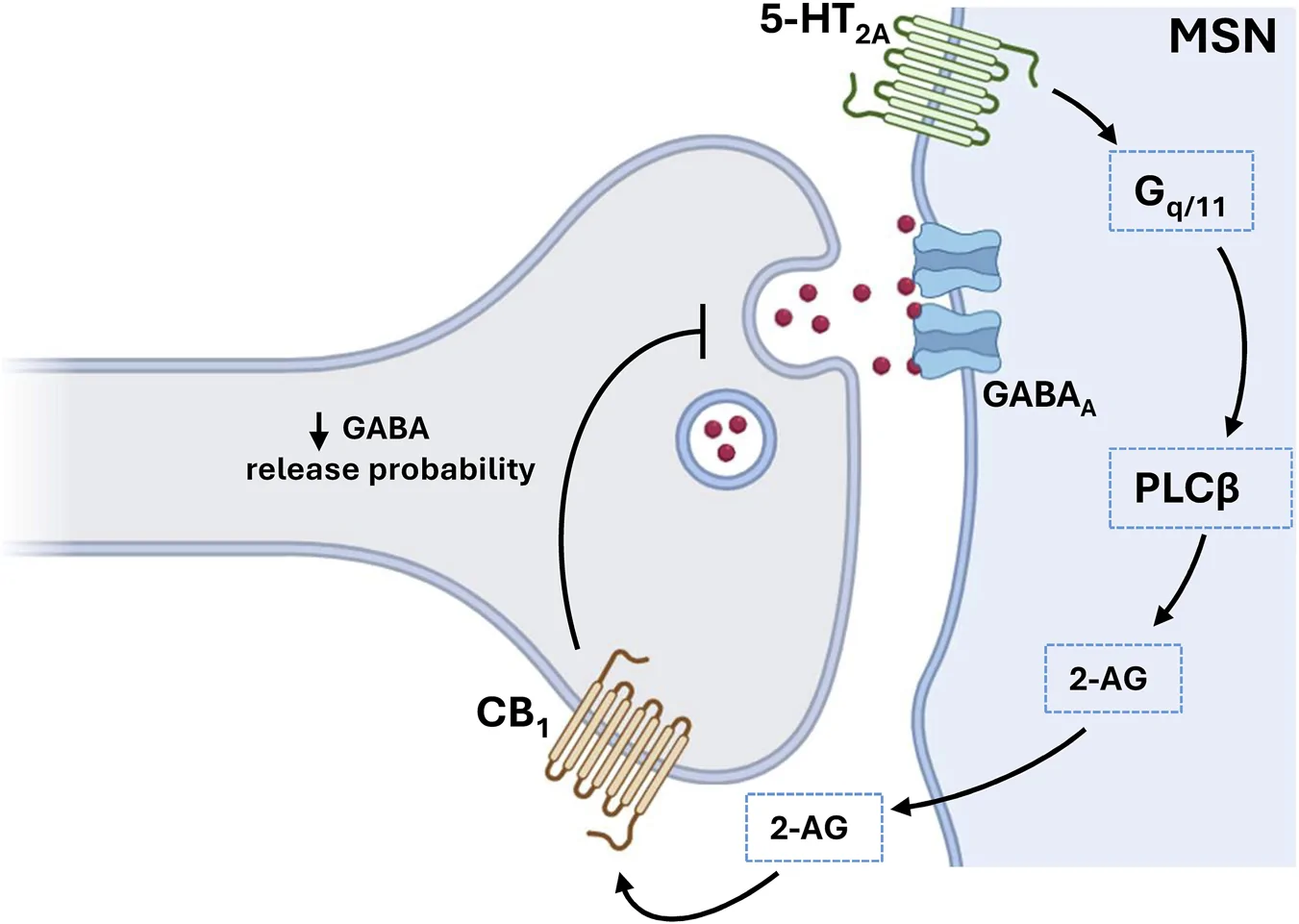
Proposed mechanism underlying 5-HT_2_A receptor-induced inhibitory long-term depression in the NAc core. Activation of postsynaptic 5-HT_2A_ receptors in MSNs engages G-protein signaling and PLCβ-dependent synthesis of the endocannabinoid 2-AG. Retrograde 2-AG signaling activates presynaptic CB_1_ receptors at GABAergic terminals, reducing GABA release probability and resulting in a presynaptically expressed iLTD of GABAergic transmission.

## 4 Discussion

In this study, we identify a previously uncharacterized mechanism by which 5-HT signaling regulates inhibitory synaptic transmission in the NAc through endocannabinoid-dependent modulation of GABAergic inputs. We demonstrate that activation of 5-HT_2A_ receptors induces an iLTD at synapses onto MSNs. This form of synaptic plasticity is expressed predominantly through a presynaptic reduction in GABA release probability. This conclusion is supported by the increase in paired-pulse ratio, modification in CV and the selective reduction in spontaneous and miniature IPSC frequency. Collectively, these findings indicate a decrease in presynaptic neurotransmitter release rather than alterations in postsynaptic GABA receptor function. These findings build upon previous studies that described eCB-dependent forms of synaptic plasticity at excitatory synapses in the NAc (Burattini et al., 2014) by demonstrating that serotonergic signaling also recruits eCB mechanisms to regulate inhibitory transmission.

Mechanistically, the data support a model in which 5-HT_2A_ receptor activation initiates a postsynaptic signaling cascade involving G_q/11_ protein activation, PLCβ engagement, and subsequent synthesis of the endocannabinoid 2-AG. Following its synthesis, 2-AG is hypothesized to function as a retrograde messenger, thereby activating presynaptic CB_1_ receptors and inducing a sustained suppression of GABA release (Chevaleyre and Castillo, 2003). The requirement for postsynaptic G-protein signaling, in conjunction with the sensitivity of this form of plasticity to PLCβ and DAG lipase inhibition, indicates that 5-HT_2A_-dependent iLTD relies on a canonical eCB biosynthetic pathway. It is noteworthy that the inhibition of PLCβ resulted in a modest reduction in iLTD, indicating the potential involvement of additional signaling mechanisms in eCB mobilization or the facilitation of CB_1_ receptor-mediated synaptic depression. However, the complete blockade of 5-HT_2A_-induced iLTD following DAG lipase inhibition provides strong support for 2-AG as the primary endocannabinoid responsible for this form of plasticity (Chevaleyre and Castillo, 2003). These findings extend previous evidence of functional connection between 5-HT_2A_ activation and retrograde cannabinoid signaling within the NAc (Best and Regehr, 2008; Burattini et al., 2014; Meza et al., 2026), providing a mechanistic framework for how neuromodulatory systems regulate inhibitory synaptic strength.

An additional level of complexity may arise from reported interactions between 5-HT_2A_ and dopamine D_2_ receptors, which can form heteroreceptor complexes in striatal regions, including the ventral striatum (Plach et al., 2019). Notably, these interactions appear to be ligand-dependent, as LSD and DOI, but not TCB-2, have been reported to enhance D_2_ receptor protomer recognition and signaling (Borroto-Escuela et al., 2014). Thus, although our findings support a mechanism initiated by 5-HT_2A_ receptor activation and subsequent recruitment of endocannabinoid signaling, whether 5-HT_2A_–D_2_ receptor interactions contribute to this form of inhibitory synaptic plasticity remains unresolved. Direct assessment of receptor proximity, together with selective manipulation of D_2_ receptor signaling, will be required to determine whether these receptor complexes play a causal role in regulating this plasticity.

From a circuit perspective, modulation of GABAergic transmission onto NAc MSNs may influence the integration of excitatory and inhibitory inputs within mesolimbic networks. A sustained reduction in GABA release mediated by 5-HT_2A_-dependent eCB signaling could alter the inhibitory control of MSNs. However, the net impact of 5-HT_2A_ signaling on NAc circuit function cannot be inferred from changes in inhibitory transmission alone. Serotonergic signaling has also been shown to induce eCB-dependent LTD at excitatory synapses in the NAc (Burattini et al., 2014), indicating that 5-HT_2A_ activation can regulate both inhibitory and excitatory synaptic transmission. Thus, rather than imposing a unidirectional shift in the excitation–inhibition balance, 5-HT_2A_-dependent eCB signaling may serve as a mechanism for dynamically tuning synaptic integration and circuit responsiveness within the NAc.

The capacity of 5-HT_2A_ receptors to modulate both excitatory and inhibitory synaptic plasticity may have significant ramifications for processes that are mediated by the NAc, including reward processing, motivation, reinforcement learning, and behavioral adaptation. The NAc undergoes extensive synaptic remodeling in response to salient experiences, stress, and exposure to addictive substances (Scofield et al., 2016). It has been hypothesized that alterations in eCB signaling may be implicated in a number of forms of maladaptive plasticity (Castillo et al., 2012; Katona and Freund, 2012). 5-HT_2A_-dependent iLTD may constitute a distinct regulatory mechanism by which serotonin modulates accumbal circuit flexibility, acting through retrograde suppression of GABAergic transmission.

## Data Availability

The raw data supporting the conclusions of this article will be made available by the authors, without undue reservation.

## Supplementary material

SUPPLEMENTARY FIGURE S1 Pharmacological validation and stability of evoked inhibitory synaptic transmission. **(A)** Representative recording showing the progressive suppression of eIPSCs during bath application of picrotoxin (PTX, 50 µM), confirming that the recorded inhibitory currents were mediated by GABA receptors. **(B)** Representative eIPSC traces obtained before and during PTX application at the time points indicated by 1 and 2 in **(A)**. **(C)** Representative eIPSC traces recorded at the time points indicated by 1 and 2 in **(B)**. **(D)** Time course of normalized eIPSC amplitude during control recordings. ACSF was bath-applied at time 0 as a control condition, and eIPSC amplitudes remained stable throughout the recording period. **(E)** Summary of paired-pulse ratio (PPR) measured at baseline and 20 min after ACSF application, showing no significant change over time. Data are presented as mean ± SEM. Within-group comparisons were performed using a paired Student’s t-test. n.s., not significant.

SUPPLEMENTARY FIGURE S2 Serotonin-induced depression of inhibitory transmission in the NAc is mediated by 5-HT_2A_ receptors. **(A)** Representative eIPSC traces recorded before (black) and after (green) application of 5-HT (10 μM) in ACSF or in the presence of the selective 5-HT_2A_ receptor antagonist MDL100907 (1 μM). **(B)** Time course of normalized eIPSC amplitude showing that bath application of 5-HT induces a long-lasting depression of inhibitory transmission in control conditions but not in the presence of MDL100907. **(C)** Summary of normalized eIPSC amplitudes demonstrating that 5-HT significantly depresses inhibitory transmission in ACSF, whereas this effect is abolished by MDL100907. Data are presented as mean ± SEM. Insets show representative traces recorded at baseline and 20 min after 5-HT application. Bar graphs show individual cells connected by lines. Within-group comparisons were performed using paired Student’s t-tests and are indicated by asterisks (*). Comparisons between experimental conditions were performed using two-way ANOVA followed by Holm–Sidak’s multiple-comparisons test and are indicated by number signs (#). ****P* < 0.001; ^#^ *P* < 0.05; n.s., not significant.
