# Supplementary material for: 5-HT2A receptor activation induces CB1-dependent presynaptic inhibitory long-term depression in the nucleus accumbens core
Source: Front Pharmacol. 2026 Oct 7;17:1916522. doi: 10.3389/fphar.2026.1916522 (PMC13683217; doi:10.3389/fphar.2026.1916522)
Supplement: Supplementary file 1 [file Table1.pdf]

Supplementary Table 1. **Pharmacological agents used in electrophysiological experiments.** Experimental concentrations, primary pharmacological targets, reported affinity or potency values, relevant selectivity and off-target considerations, and the experimental basis for the selected concentrations are summarized for each compound. Affinity and potency values are reported as *K*i when receptor-binding data are available; for compounds that do not primarily act as receptor ligands, the corresponding *K*d or IC_{50} values are provided. N/A indicates that a receptor-binding affinity constant is not applicable.

Pharmacological agent	Concentration used	Primary target/action	Reported affinity or potency	Relevant selectivity / off-target considerations	Basis for experimental concentration
**5-HT**	10 µM, 1 min in bath	Endogenous agonist at 5-HT receptors	*K*i is receptor subtype- and assay-dependent; for 5-HT, reported *K*i = 6.0–8.4 (human) and 6.0–6.5 (rat)	Endogenous non-selective serotonergic agonist	Concentrations in the micromolar range are commonly used to assess serotonergic modulation in brain-slice electrophysiology (Araneda and Andrade, 1991; Zhang, 2003; Meza et al., 2026)
**TCB-2**	10 µM, 1 min in bath	5-HT_{2A} receptor agonist	*K*i = 0.73 nM (rat); 0.75 nM (human)	High-affinity 5-HT_{2A} agonist; the complete off-target binding profile has not been fully characterized	1–10 µM has precedent in ex vivo electrophysiological studies of 5-HT_{2A} -dependent signaling (Austgen and Kline, 2013; Meza et al., 2026)
**MDL100907**	1 µM, in bath	5-HT_{2A} receptor antagonist	*K*i = 0.36 nM	>80-fold selective for 5-HT_{2A} over other serotonergic receptor subtypes	1 µM has been used to block 5-HT_{2A} -mediated responses in neuronal electrophysiology (Meza et al., 2026)
**WIN55,212-2**	5 µM, in bath	CB_1/CB_2 receptor agonist	*K*i = 62.3 nM CB_1; 3.3 nM CB_2 in human cloned receptors	Non-selective cannabinoid receptor agonist; affinity values are assay-dependent	1–5 µM produces robust cannabinoid receptor-mediated suppression of synaptic transmission in acute brain slices (Guiffa-Gómez et al., 2026; Meza et al., 2026)
**AM251**	10 µM, in bath	CB_1 receptor antagonist/inverse agonist	*K*i = 7.49 nM	306-fold selectivity for CB_1 over CB_2; also reported to act at GPR55	1–10 µM concentrations are commonly used for CB_1 blockade in acute brain slices (Morales-Weil et al., 2020; Meza et al., 2026)
**GDPβS**	2 mM, intracellular	Non-hydrolysable GDP analogue; inhibits G-protein signaling	N/A — not a receptor ligand	Intracellular nucleotide analogue that interferes with GPCR-dependent G-protein activation	1–2 mM intracellular GDPβS has been used in whole-cell recordings to test postsynaptic G-protein dependence (Morales-Weil et al., 2020; Meza et al., 2026)
**U73122**	20 µM, intracellular	PLC inhibitor	IC_{50} = 1–5 µM in PLC-dependent platelet aggregation assays	Not completely selective; also modulates TRPM4 and TRPM3 channels	Intracellular U73122 has been used in whole-cell neuronal recordings to inhibit PLC-dependent signaling (Choi et al., 2005)
**RHC80267**	30 µM, intracellular	Diacylglycerol lipase inhibitor	IC_{50} = 1.1 µM in rat cardiac myocytes; 4 µM in canine platelets	Weakly inhibits PLC and PLA_2; cholinesterase inhibition has also been reported	30 µM has been used experimentally to inhibit DAGL-dependent endocannabinoid synthesis (Zhong et al., 2015)
**TTX**	500 nM, in bath	Voltage-gated Na^+ channel blocker	*K*d = 1.8 nM in rat brain membranes	Blocks TTX-sensitive Na_v channels; TTX-resistant isoforms have substantially lower sensitivity	500 nM is routinely used to eliminate action potential-dependent synaptic transmission (Guiffa-Gómez et al., 2026)
**D-AP5 (D-APV)**	25 µM, in bath	Competitive NMDA receptor antagonist	*K*d = 1.4 µM	Selective competitive antagonist at the glutamate-binding site of NMDA receptors	25 µM D-APV is routinely used to suppress NMDA receptor-mediated transmission during IPSC recordings (Santander et al., 2025; Guiffa-Gómez et al., 2026; Meza et al., 2026)
**CNQX**	20 µM, in bath	AMPA/kainate receptor antagonist	IC_{50} = 0.3 µM (AMPA); 1.5 µM (kainate)	At higher concentrations, CNQX also antagonizes the glycine modulatory site of NMDA receptors $IC_{50} \approx 25$ µM	20 µM CNQX is commonly used to suppress AMPA/kainate transmission during IPSC recordings (Santander et al., 2025; Guiffa-Gómez et al., 2026; Meza et al., 2026)
**Picrotoxin (PTX)**	50 µM, in bath	Non-competitive $GABA_A$ receptor channel blocker	IC_{50} = ~1–10 µM has been reported for recombinant $GABA_A$	Blocks the picrotoxin-sensitive chloride channel associated with $GABA_A$ receptors; potency depends on receptor subunit composition and receptor activation state	50 µM provides robust blockade of $GABA_A$ receptor-mediated currents and was used here to pharmacologically verify the GABAergic nature of the recorded eIPSCs (Guiffa-Gómez et al., 2026)
